# Effectiveness of Pharmacist–Physician Collaborative Management for Patients With Idiopathic Pulmonary Fibrosis Receiving Pirfenidone

**DOI:** 10.3389/fphar.2020.529654

**Published:** 2020-11-26

**Authors:** Yukari Satsuma, Hiroaki Ikesue, Kaori Kusuda, Mami Maeda, Nobuyuki Muroi, Ryobu Mori, Mariko Kogo, Ryosuke Hirabayashi, Kazuma Nagata, Atsushi Nakagawa, Ryo Tachikawa, Keisuke Tomii, Tohru Hashida

**Affiliations:** ^1^ Department of Pharmacy, Kobe City Medical Center General Hospital, Kobe, Japan; ^2^ Department of Respiratory Medicine, Kobe City Medical Center General Hospital, Kobe, Japan

**Keywords:** idiopathic pulmonary fibrosis, pirfenidone, pharmacist, physician, collaborative management, ambulatory care pharmacy practice

## Abstract

**Background:** Pirfenidone is an anti-fibrotic agent used to treat patients with idiopathic pulmonary fibrosis (IPF). Managing adverse drug events and ensuring compliance with pirfenidone treatment for a prolonged period are important to reduce the rate of disease progression. To maximize the benefits of pirfenidone treatment, we established and evaluated an ambulatory care pharmacy practice, a model of pharmacist–physician collaborative management, for patients receiving pirfenidone.

**Methods:** We conducted a retrospective chart review of 76 consecutive patients treated with pirfenidone in the Kobe City Medical Center General Hospital, Japan, between January 2012 and January 2019. The first group (61 patients) received pirfenidone treatment as conventional management, whereas the second group (15 patients) started pirfenidone based on collaborative pharmacist–physician management. The drug discontinuation rate and time to drug discontinuation were compared between the groups. To analyze factors associated with pirfenidone discontinuation, we used a multivariate Cox regression analysis to evaluate the baseline characteristics of patients, including those receiving the collaborative management. Clinical outcomes were compared using a propensity score matched analysis.

**Results:** In the collaborative management group, pharmacists made 56 suggestions, including suggestions for supportive care (51 suggestions), to the physicians. Among these suggestions, 52 were accepted by the physicians. The discontinuation rates at 3 [6.7% (1/15) vs. 26.2% (16/61)] and 6 [9.1% (1/11) vs. 36.1% (22/61)] months were lower in the collaborative management group than in the conventional management group. Multivariate analysis revealed that collaborative management [hazard ratio (HR) 0.34, 95% CI 0.08–0.96, *p* = 0.041] and predicted baseline forced vital capacity <60% (HR 2.13, 95% CI 1.17–3.85, *p* = 0.015) were significantly associated with pirfenidone discontinuation. The time to drug discontinuation was also significantly longer in the collaborative management group than in the conventional management group (*p* = 0.034, log-rank test). Propensity score matched analysis confirmed a significant correlation between collaborative management and drug discontinuation time (HR 0.20, 95% CI 0.03–0.84, *p* = 0.027).

**Conclusions:** We established an ambulatory care pharmacy practice for out-patients with IPF receiving pirfenidone. The results suggest that collaborative management may help prevent pirfenidone discontinuation compared with conventional management.

## Introduction

Idiopathic pulmonary fibrosis (IPF), the most common form of idiopathic interstitial pneumonia (IIP), is a progressive lung disease characterized by chronic refractory cough, shortness of breath, and exercise limitation (Ley et al., 2011; Lederer and Martinez, 2018). The prognosis of patients with IPF is poor, with a median survival of 2–5 years after diagnosis (Ley et al., 2011; Raghu et al., 2015; Homma et al., 2018; Lederer and Martinez, 2018). Two anti-fibrotic agents, pirfenidone and nintedanib, are currently approved for the treatment of IPF in several countries (Raghu et al., 2015; Homma et al., 2018). The clinical efficacy of pirfenidone in patients with IPF has been confirmed in clinical trials in Japan (Azuma et al., 2005; Taniguchi et al., 2010) and in multinational randomized clinical trials (Noble et al., 2011; King et al., 2014). Continuing pirfenidone for a prolonged period is important to reduce the rate of disease progression. The most common adverse drug events (ADEs) associated with pirfenidone are gastrointestinal tract- and skin-related events (Lancaster et al., 2016; Noble et al., 2016). The management of ADEs is essential because these symptoms can lead to discontinuation of pirfenidone treatment by patients (Ogura et al., 2015; Hughes et al., 2016; Lancaster et al., 2017; Cottin et al., 2018).

Studies have reported the usefulness of patient-centered, multidisciplinary team care, which provides support, education, and empowerment to patients with IPF receiving pirfenidone (Duck et al., 2015; Ferrara et al., 2018). We assembled a multidisciplinary IIP support team comprising healthcare professionals, including physicians, pharmacists, nurses, dietitians, physical therapists, and social workers, to support patients with IIP. To support the functions of this team, we established an ambulatory care pharmacy practice to provide pharmacist–physician collaborative management of out-patients with IPF receiving anti-fibrotic therapy in September 2017. The main purpose of the collaborative management was to prevent early discontinuation of anti-fibrotic agents. Therefore, pharmacists provided information to physicians regarding appropriate prescriptions and education to patients to minimize ADEs and enhance adherence to anti-fibrotic agents. In the present study, we evaluated the usefulness of this management system for patients receiving pirfenidone.

## Materials and Methods

### Establishing Pharmacist–Physician Collaborative Management of Pirfenidone Treatment for Out-Patients With Idiopathic Pulmonary Fibrosis

We constructed a multidisciplinary IIP support team composed of healthcare professionals, including physicians, pharmacists, nurses, dietitians, physical therapists, and social workers, in the Kobe City Medical Center General Hospital, Japan. Initially, we prepared a patient education book, which helped to develop consensus among the healthcare professionals regarding the treatment and care of patients with IPF and to provide education to patients. We then established an ambulatory care pharmacy practice, comprising the pharmacist–physician collaborative management of out-patients with IPF receiving antithrombotic therapy, in September 2017. The objectives were to provide information to physicians for appropriate prescriptions and to patients to minimize ADEs and drug–drug interactions, to enhance the adherence of patients to treatment and maximize the effectiveness of anti-fibrotic therapies.

**FIGURE 1:**
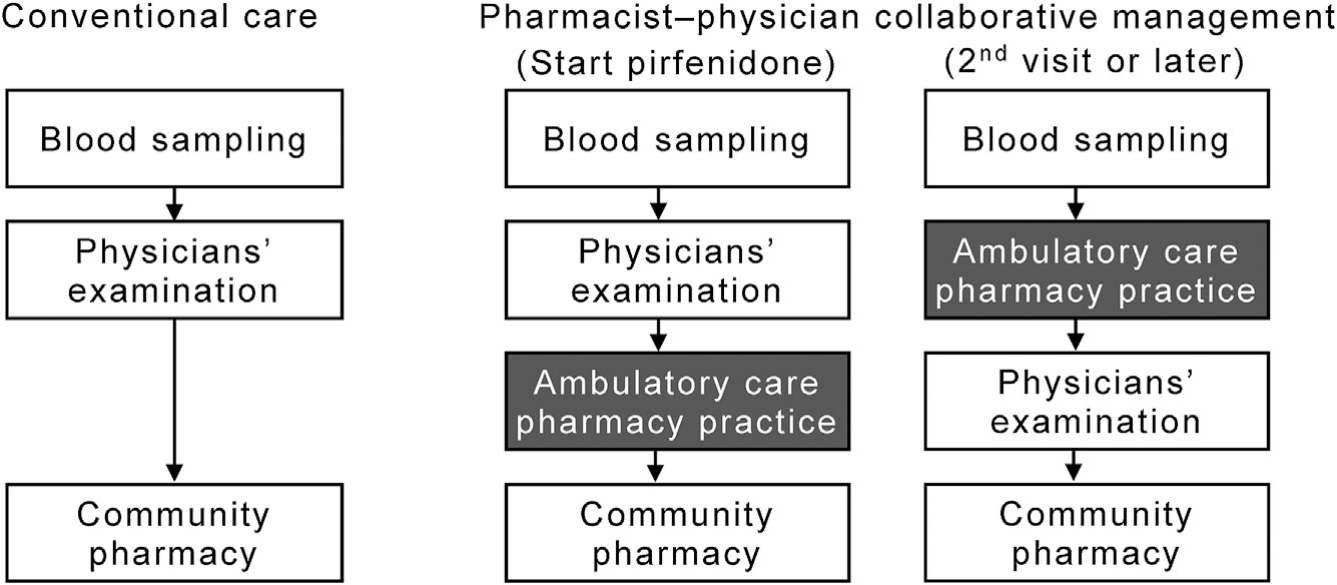
Flow diagram of patients in the pharmacist–physician collaborative management and conventional groups. The collaborative management was initiated in September 2017; thereafter, 15 consecutive patients (the collaborative management group) were collaboratively managed by physicians and pharmacists. From January 2015 to August 2017, 61 patients received conventional care with pirfenidone (the conventional group).

A patient flow diagram, including the ambulatory care pharmacy practice, is shown in Figure 1. When pirfenidone was initially prescribed, the physician advised the patient to visit the ambulatory care pharmacy practice after their clinical examination. In the ambulatory drug consultation room, a pharmacist educated the patients using the patient education book as follows: 1) initial dosage and time of dose; 2) standard titration schedule of pirfenidone; 3) symptoms and management of pirfenidone-associated ADEs, such as gastrointestinal events and photosensitivity; and 4) drug–drug interactions. The pharmacist also queried the patient about concomitant medications and supplements to avoid any drug–drug interactions. At the second visit or later, the patient visited the pharmacy service before being examination by the physician. The pharmacist assessed: 1) any ADEs and their grade according to the National Cancer Institute Common Terminology Criteria for Adverse Events version 4.0; 2) whether the patient was able to take the medication for ADEs appropriately; 3) changes in any concomitant medications or supplements; and 4) adherence to pirfenidone, which was determined by asking the patient how many of the prescribed tablets remained. The pharmacist repeatedly educated the patient about the management of ADEs. The patient was allowed to ask the pharmacist any questions directly in the ambulatory care pharmacy practice or by telephone. Per requirement, based on their assessment, the pharmacist suggested prescriptions to the physician. The patient then received the medication, including pirfenidone, from the community pharmacies.

The ambulatory care pharmacy practice comprised three clinical pharmacists who also provided clinical pharmacy service for in-patients in clinical wards. Our pharmacy practice was provided for all patients for the first 3 months after the start of pirfenidone therapy. If the patients wished to continue the service, they could visit the ambulatory care pharmacy practice any time before being examined by a physician. The ambulatory care pharmacy practice usually required 20 min per outpatient for a face-to-face consultation and an additional 5 min to enter the medical chart into an electronic medical record system to share the patients’ information with the IPF support team.

### Patients

We conducted a retrospective chart review of 76 consecutive Japanese patients with IPF prescribed pirfenidone between January 2015 and January 2019. All subjects received pulmonary care in the Kobe City Medical Center General Hospital, Japan. Pharmacist–physician collaborative management of patients receiving pirfenidone treatment was initiated in September 2017; thereafter, 15 consecutive patients (the collaborative management group) were managed collaboratively by physicians and pharmacists. From January 2015 to August 2017, 61 patients received conventional care with pirfenidone (the conventional group). Patients aged at least 20 years, diagnosed with pulmonary fibrosis by their primary pulmonologist during routine clinical care, and prescribed pirfenidone were included. One patient who received perioperative pirfenidone only (Iwata et al., 2016) was excluded. This study was conducted in accordance with the Declaration of Helsinki. The study protocol was approved by the Institutional Review Board of the Kobe City Medical Center General Hospital (No. h190401).

In Japan, the approved dosage of pirfenidone is 1,800 mg/day (Azuma et al., 2005; Homma et al., 2018). In accordance with the package insert of pirfenidone in Japan, one 200 mg pirfenidone tablet is administered orally three times a day (t.i.d.) after meals for the first 2 weeks (600 mg/day), two tablets t.i.d. for the next 2 weeks (1,200 mg/day), and three tablets t.i.d. thereafter (1,800 mg/day). Physicians adjusted the dose-escalation schedule by observing each patient’s condition. In the conventional care group, supportive care medicines, including domperidone, metoclopramide, and mosapride, were prescribed at the physicians' discretion. In contrast, all patients in the collaborative management group were prescribed domperidone 5 mg t.i.d. orally at the onset of pirfenidone therapy (Costabel et al., 2014); the subsequent domperidone dose was prescribed at the physicians’ discretion based on suggestions made by the pharmacists.

### Outcome Measures and Data Collection

The primary objective of this study was to evaluate the effect of pharmacist–physician collaborative management on pirfenidone discontinuation in patients with IPF. The secondary objectives included the adherence rate and suggestions made to physicians by the pharmacists in the collaborative management group. To assess adherence to pirfenidone, the pharmacists queried patients about the remaining number of pirfenidone tablets at each visit for the first 3 months after the start of treatment. The adherence rate (%) was calculated as follows (number of tablets taken)/(number of tablets prescribed) × 100. We also evaluated factors associated with pirfenidone discontinuation owing to ADEs in the conventional management group. All data were collected from the electronic medical records. The data cut-off date was January 31, 2019.

### Statistics

Categorical data are displayed as the number of patients (n) and the respective relative frequencies. Values were compared using Fisher’s exact test. For continuous data, median [interquartile range (IQR)] values are presented. Differences between groups were analyzed using Wilcoxon rank sum test. To analyze factors associated with pirfenidone discontinuation, a single predictor Cox regression analysis was performed using patients’ age, sex, predicted baseline forced vital capacity (FVC), body mass index (BMI), smoking status, and pharmacist–physician collaboration management as independent variables. Significant factors in the univariate analysis were evaluated as potential covariates in the subsequent multivariable Cox regression analysis. The time to drug discontinuation was estimated using the Kaplan-Meier method and compared using the log-rank test. Patients who were still taking pirfenidone and were free from progression were censored at the last follow-up.

To adjust for the other baseline factors, a sensitivity analysis was conducted by propensity score matching. We estimated the propensity score by modeling the probability of being in the conventional management group vs. the collaborative management group. The following variables were included in the regression model: age, body weight, smoking status, concomitant use of prednisolone, FVC, and DLco. A 1:1 matching (without replacement) in the two treatment groups was achieved using the nearest neighbor method with a 0.25-width caliper of SD of the logit of propensity scores to reduce bias by these potential confounding factors. The matched data were analyzed to confirm the robustness of the primary analysis results. Data were analyzed using JMP 13.2.1 (SAS Institute Inc., Cary NC, United States). The results with a *p*-value of <0.05 were considered statistically significant.

## Results

### Baseline Characteristics of Patients

Between January 2015 and August 2017, 61 consecutive patients with IPF were prescribed pirfenidone treatment (the conventional group). After establishing the ambulatory care pharmacy practice, between September 2017 and January 2019, 15 consecutive patients with IPF were prescribed pirfenidone (the collaborative management group). The baseline characteristics of the 74 eligible patients are summarized in Table 1. There were no significant differences in the proportions of patients who were male (80.0 vs. 54.2%, *p* = 0.138), ex-smokers (73.3 vs. 54.2%, *p* = 0.245), using home oxygen therapy (13.3 vs. 11.9%, *p* = 1.000), or concomitantly receiving prednisolone (46.7 vs. 32.2%, *p* = 0.367) between the collaborative management and conventional groups. Similarly, baseline age, body weight, BMI, FVC, and 6-min walk distance were not significantly different between the groups. The median (IQR) predicted FVC (%) in the collaborative management and conventional groups was 71.8 (64.4–85.0) and 67.6 (53.8–85.0), respectively. The proportion of patients with a predicted FVC of <60% was not different between the groups [20.0% (3/15) vs. 37.3% (22/59), *p* = 0.240]. The baseline values of predicted diffusing capacity of carbon monoxide (DLco, %) were not evaluated in 24 and three patients, in the conventional and the collaborative management group, respectively. The median (IQR) predicted DLco (%) of the remaining patients was significantly lower in the collaborative management group than in the conventional group [51.8 (42.5–62.2) vs. 63.3 (56.3–85.0), *p* = 0.031].

**TABLE 1:** Patient characteristics.

	Collaborative management (n = 15)	Conventional (n = 61)	*P*-value
Age, years	76 (71–80)	74 (68–80)	0.414
Male, n (%)	12 (80.0)	34 (55.7)	0.139
Body weight, kg	58.5 (51.5–65.0)	57.6 (49.0–66.0)	0.548
BMI, kg/m^2^	22.7 (19.8–24.8)	22.3 (20.2–24.1)	0.912
Smoker, n (%)			
Ex	11 (73.3)	34 (55.7)	0.254
Never	4 (26.7)	27 (44.3)	
Home oxygen therapy, n (%)	2 (13.3)	7 (11.5)	1.000
Prednisolone use, n (%)	7 (46.7)	20 (32.8)	0.372
Pulmonary function tests			
FVC (L)	2.3 (1.6–2.7)	1.9 (1.5–2.5)	0.464
FVC (% pred)	71.8 (64.4–85.0)	67.6 (53.8–87.0)	0.676
DLco (% pred)	51.8 (42.5–62.2)^a^	63.3 (56.3–85.0)^b^	0.031
6-min walk distance, m	413 (256–485)^c^	445 (375–500)^d^	0.280

DLco, diffusing capacity of carbon monoxide; FVC, forced vital capacity. Continuous data are shown as the median (interquartile range). Fisher’s exact test and Wilcoxon rank sum tests were used to compare categorical and continuous data, respectively.

a Missing data from 3 patients.

b Missing data from 24 patients.

c Missing data from 1 patient.

d Missing data from 14 patients.

### Effect of Pharmacist–Physician Collaboration Management on Pirfenidone Discontinuation

Pirfenidone discontinuation rates at 3, 6, and 12 months are shown in Supplementary Figure S1. In the collaborative management group, the observation period for four and six patients was less than 6 and 12 months, respectively. Three patients discontinued pirfenidone owing to ADEs (grade 2 photosensitivity at 2 months, grade 2 rash at 9 months, and grade 1 photosensitivity at 11 months) within the first 12 months. In contrast, among the 61 patients in the conventional group, 21 discontinued pirfenidone owing to ADEs (13 patients, nausea or anorexia; 3, malaise; 2, hepatotoxicity; and 3, other events) and 10 patients discontinued owing to an acute exacerbation of IPF or disease progression, within the first 12 months. One patient was transferred to another hospital after 11.3 months of treatment. The rates of discontinuation at 3 [6.7 (1/15) vs. 26.2% (16/61), *p* = 0.167] and 6 [9.1 (1/11) vs. 36.1% (22/61), *p* = 0.093] months were lower in the collaborative management group than in the conventional group; however, the difference was not significant.

**FIGURE 2:**
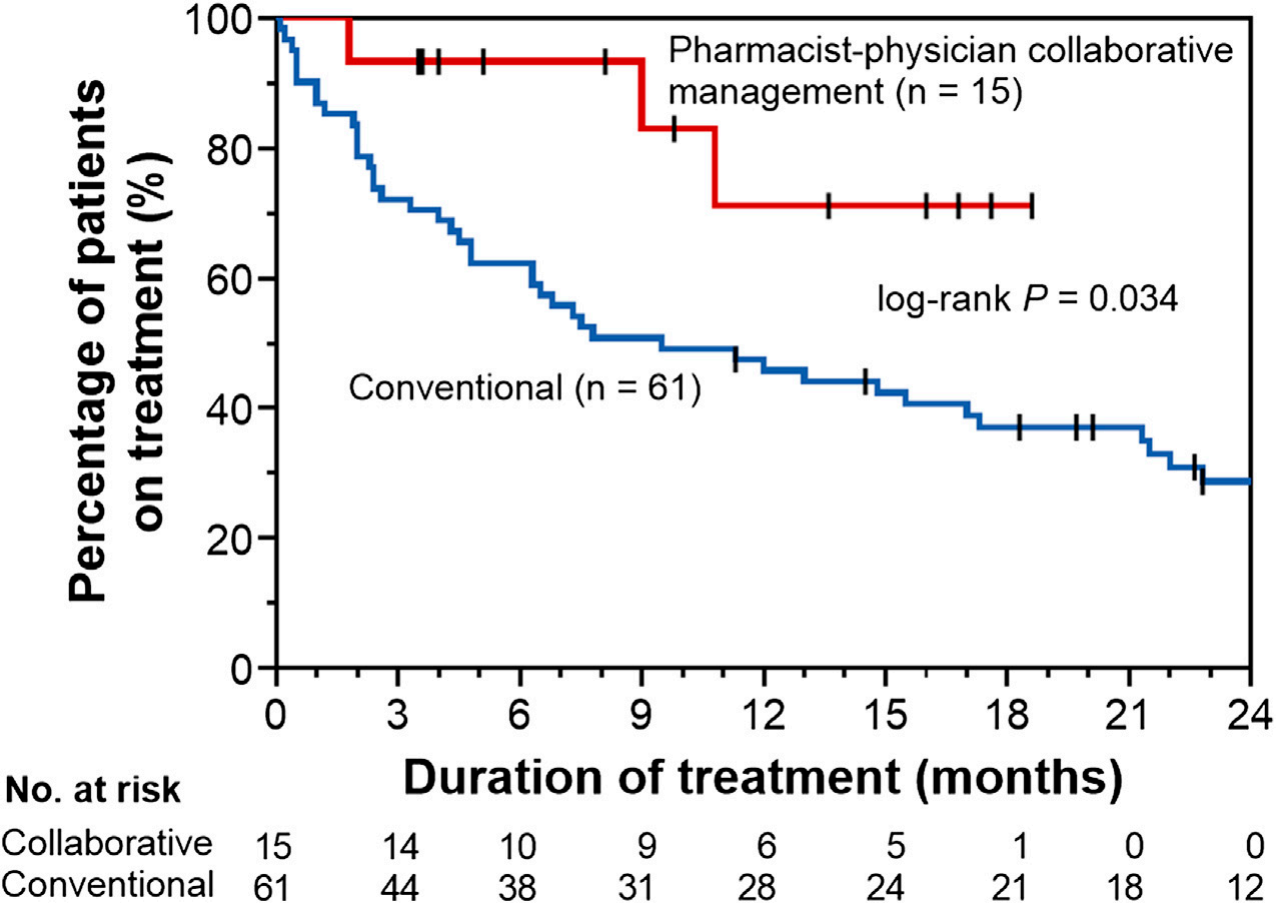
Kaplan–Meier curves for time to drug discontinuation in the pharmacist–physician collaborative management and conventional groups.

To evaluate the effect of pharmacist–physician collaborative management on the discontinuation of pirfenidone, we conducted univariate and multivariate analyses using a Cox proportional hazards model. The results revealed that collaborative management [hazard ratio (HR) 0.34, 95% CI 0.08–0.96, *p* = 0.041] and a predicted baseline FVC of <60% (HR 2.13, 95% CI 1.17–3.85, *p* = 0.015) were significantly associated with pirfenidone discontinuation (Table 2). The Kaplan–Meier curve for the continuation of pirfenidone therapy is shown in Figure 2. The time to drug discontinuation was significantly longer in the collaborative management group than in the conventional group (*p* = 0.034, log-rank test). In contrast, the prescription of antiemetics did not significantly decrease the rate of pirfenidone discontinuation owing to ADEs in the conventional group (Supplementary Table S1).

**TABLE 2:** Univariate and multivariate Cox regression models for drug discontinuation.

Factor	Univariate analysis		Multivariate analysis			
	HR, (95% CI)	*P*-value	HR, (95% CI)	*P*-value		
Collaborative management	0.30 (0.07–0.84)	0.018	0.34 (0.08–0.96)	0.041		
Predicted baseline FVC <60%	2.32 (1.27–4.18)	0.007	2.13 (1.17–3.85)	0.015		
Age >70 years	0.96 (0.52–1.86)	0.896	—	—		
Female sex	1.16 (0.63–2.08)	0.631	—	—		
BMI, kg/m^2^	1.04 (0.96–1.12)	0.387	—	—		
Ex-smoker	0.98 (0.55–1.78)	0.953	—	—		
Home oxygen therapy	1.26 (0.43–2.91)	0.642	—	—		
Concomitant use of prednisolone	1.61 (0.87–2.89)	0.127	—	—		

BMI, body mass index; FVC, forced vital capacity; HR, hazard ratio.

We also performed propensity score matching to balance patient characteristics between the collaborative management and conventional groups. There were no significant differences in the factors between the two groups (Table 3). In the propensity score-matched cohorts, the median time to drug discontinuation was significantly longer in the collaborative management group than in the conventional group [not reached (NR) (95% CI 1.8–NR) vs. 2.9 months (95% CI 0.1–NR), *p* = 0.028, log-rank test], and there was a significant association between collaborative management and time to drug discontinuation (HR 0.20, 95% CI 0.03–0.84, *p* = 0.027).

**TABLE 3:** Patient characteristics after propensity score matching.

	Collaborative management (n = 10)	Conventional (n = 10)	*P*-value
Age, years	76 (68–79)	78 (71–83)	0.425
Male, n (%)	7 (70.0)	5 (50.0)	0.650
Body weight, kg	59.1 (52.6–68.8)	52.9 (50.4–65.1)	0.427
BMI, kg/m^2^	23.4 (19.9–24.9)	21.8 (20.5–23.6)	0.677
Smoker, n (%)			
Ex	7 (70.0)	7 (70.0)	1.000
Never	3 (30.0)	3 (30.0)	
Home oxygen therapy, n (%)	1 (10.0)	1 (10.0)	1.000
Prednisolone use, n (%)	5 (50.0)	5 (50.0)	0.372
Pulmonary function tests			
FVC (L)	2.4 (1.6–2.8)	2.2 (1.8–2.4)	0.520
FVC (% pred)	77.1 (69.1–85.3)	76.5 (53.7–88.0)	1.000
DLco (% pred)	58.7 (47.5–79.0)	54.2 (37.9–67.1)	0.473
6-min walk distance, m	450 (276–569)^a^	410 (272–488)^b^	0.773

DLco, diffusing capacity of carbon monoxide; FVC, forced vital capacity. Continuous data are shown as the median (interquartile range). Fisher’s exact test and Wilcoxon rank sum tests were used to compare categorical and continuous data, respectively.

a Missing data from 1 patient.

b Missing data from 2 patients.

### Activities of Pharmacists in the Ambulatory Care Pharmacy Practice

In the collaborative management group, the pharmacists made a total of 56 suggestions to the physicians (Table 4). Among these suggestions, the required number of supportive care medicines that were previously prescribed (38 suggestions) was the most frequent. The suggestions also comprised additional supportive care (13 suggestions), including antiemetics or photosensitivity prevention, and others (5 suggestions). Among the 56 suggestions, 52 (92.9%) were accepted by the physicians.

**TABLE 4:** Number of suggestions provided by the pharmacists and physicians’ responses.

	Number of suggestions	Number of suggestions accepted by physicians
Required number of supportive care medicines that were previously prescribed^a^	38	35 (92.1%)
Additional supportive care	13	13 (100%)
Others	5	4 (80.0%)

a In pharmacist–physician collaborative management, the pharmacists assessed whether the patients could appropriately take supportive care medications, such as domperidone. If the patient took the supportive medicine inadequately, the pharmacist educated the patient on the appropriate use. Then, the pharmacist suggested the number of required supportive care medicines to the physicians.

The pharmacists assessed patients’ adherence to pirfenidone at each visit to the ambulatory care pharmacy practice. The results revealed that the median adherence of patients to pirfenidone for the first 3 months was 99.4% (98.4–100%).

## Discussion

We established and evaluated an ambulatory care pharmacy practice for the pharmacist–physician collaborative management of out-patients with IPF receiving anti-fibrotic therapy. Of the 56 suggestions made by the pharmacists to the physicians, 52 (92.9%) were accepted by the physicians. The rates of pirfenidone discontinuation owing to ADEs after 3 and 6 months of treatment tended to be lower after the initiation of the ambulatory care pharmacy practice, and the time to pirfenidone discontinuation in the collaborative management group was significantly longer than that in the conventional management group. Moreover, multivariate analysis revealed that the collaborative management approach significantly reduced the risk of pirfenidone discontinuation. This result was confirmed by a propensity score-matching analysis. To the best of our knowledge, this is the first report to demonstrate that pharmacist–physician collaborative management in an ambulatory setting successfully prevented the discontinuation of pirfenidone treatment in patients with IPF.

In real-world clinical practice, the rates of pirfenidone discontinuation owing to any reason and owing to ADEs at 12 months have been reported to be 35–75% (Ogura et al., 2015; Bando et al., 2016; Barratt et al., 2018; Ogawa et al., 2018; Uehara et al., 2018; Sköld et al., 2019) and 15–35% (Ogura et al., 2015; Bando et al., 2016; Galli et al., 2017; Barratt et al., 2018), respectively. In our study, the rates of pirfenidone discontinuation owing to any reason and owing to ADEs at 12 months were 51.7 vs. 33.3% and 35.0 vs. 33.3% in the conventional vs. collaborative management groups; these values were generally within the range reported previously. The multivariate analysis revealed that an FVC <60% was a risk factor for the discontinuation of pirfenidone. This result supported the findings of previous studies (Galli et al., 2017; Barratt et al., 2018; Uehara et al., 2018). On the contrary, a part of our results was not consistent with the results of previous larger-scale studies. In the PASSPORT study, female sex was a significant risk factor for early treatment discontinuation due to ADEs (Cottin et al., 2018). The percentage of females was relatively higher in the conventional group than in the collaborative management group. Although we performed propensity score matching to adjust the imbalance in patient characteristics between the groups, it was difficult to completely adjust these imbalances. This was likely due to the small sample size in our study; therefore, our results should be confirmed in larger-scale studies.

The usefulness of patient-centered, multidisciplinary team care for patients with IPF has been previously assessed (Chaudhuri et al., 2014; Wuyts et al., 2014; Duck et al., 2015; Martinez and Flaherty, 2017). Duck et al. reported the effective collaboration of specialist IPF nurses with physicians and patients in the IPF Care program (Duck et al., 2015). In their review article, they described that nurses provide several supports. The IPF Care program, which is initiated when the patient is prescribed pirfenidone, involves phone calls and patient-tailored information booklets. Additionally, the nurses discuss the treatment of each patient directly with the physicians. In some countries, the program also involves face-to-face visits with nurses. In our hospital, nurses have multiple important roles within the multidisciplinary IIP support team. For example, nurses advise patients and their family about their lifestyle, provide education about home oxygen therapy, and support advanced care planning processes (Rajala et al., 2016). Additionally, we have previously provided ambulatory care pharmacy practice services for patients treated with oral anticancer drugs and direct-acting antiviral agents for hepatitis C virus (Yamamoto et al., 2018). Therefore, based on our experience, we established the ambulatory care pharmacy practice for patients receiving anti-fibrotic agents.

In our ambulatory care pharmacy practice, the pharmacists educated patients on how to take supportive care medicines upon initiation of pirfenidone treatment. At the second visit or later, the pharmacists assessed the potential ADEs and the appropriateness of the supportive care medicines; they then suggested prescriptions to the physicians. Most of the suggestions made by the pharmacists pertained to the required number of supportive care medicines that were previously prescribed (38/56 suggestions). Interestingly, although 52 of the 61 patients in the conventional care group were prescribed antiemetics, including domperidone, metoclopramide, and mosapride, the incidence of drug discontinuation owing to ADEs in these patients was not significantly decreased (Supplementary Table S1). Overall, prescribing supportive care medications alone might not be sufficient to manage ADEs, but detailed explanations are essential for each patient. Numerous studies have reported the usefulness of pharmacist-involved team care in pharmacotherapies (Yamada and Nabeshima, 2015; Yamamoto et al., 2018; Crutzen et al., 2019; Zhang et al., 2019). Our results showed that pharmacist–physician collaborative management, in an ambulatory care setting, can also improve care for patients with IPF receiving pirfenidone.

There were some limitations to our study. First, this was a single-centered, retrospective study. Second, the number of patients in the pharmacist–physician collaborative management group was small, and the sample sizes were imbalanced between the groups. Despite performing propensity-score matching, in addition to the primary analysis, to confirm the robustness of the usefulness of our pharmacist–physician collaborative management, the sample size of the score-matched cohort was very small. Our preliminary findings should be confirmed prospectively in further studies in other institutions. Thirdly, the ideal follow-up period could not be determined from this study design. Our pharmacy practice was generally provided for all patients during the first 3 months from the start of pirfenidone treatment because the majority of dose reductions or discontinuations are observed during the early phase (Lancaster et al., 2017). However, two patients in the collaborative management group discontinued pirfenidone owing to ADEs more than 3 months after treatment initiation.

In conclusion, we established an ambulatory care pharmacy practice for the pharmacist–physician collaborative management of out-patients with IPF receiving pirfenidone treatment. The results of our study suggest that, compared to conventional management, collaborative management may be useful for preventing the discontinuation of pirfenidone. Our preliminary findings warrant further prospective investigations with a larger sample size.

## Data Availability Statement

The datasets generated for this study are available on request to the corresponding author.

## Ethics Statement

The studies involving human participants were reviewed and approved Institutional Review Board, Kobe City Medical Center General Hospital, Kobe, Japan.

## Author Contributions

YS, HI, KK, RM, MK, RT, KT, and TH conceived and designed the study. YS, HI, KK, MM, RM, and MK collected the data. HI analyzed data. YS, KK, and MM verified the analyzed data. YS drafted the manuscript. HI, NM, RM, MK, RH, KN, AN, RT, KT, and TH revised the manuscript. KK, NM, RM, MK, RH, KN, AN, RT, KT, and TH interpreted data. All authors reviewed and approved the final manuscript.

## Conflict of Interest

KT received lecture fee from Shionogi and Co., Ltd.

The remaining authors declare that the research was conducted in the absence of any commercial or financial relationships that could be construed as a potential conflict of interest.

## References

[B1] Azuma A. Nukiwa T. Tsuboi E. Suga M. Abe S. Nakata K. (2005). Double-blind, placebo-controlled trial of pirfenidone in patients with idiopathic pulmonary fibrosis. Am. J. Respir. Crit. Care Med. 171, 1040–1047. 10.1164/rccm.200404-571OC 15665326

[B2] Bando M. Yamauchi H. Ogura T. Taniguchi H. Watanabe K. Azuma A. (2016). Clinical experience of the long-term use of pirfenidone for idiopathic pulmonary fibrosis. Intern. Med. 55 (5), 443–448. 10.2169/internalmedicine.55.5272 26935361

[B3] Barratt S. L. Mulholland S. Al Jbour K. Steer H. Gutsche M. Foley N. (2018). South-West of England’s experience of the safety and tolerability pirfenidone and nintedanib for the treatment of idiopathic pulmonary fibrosis (IPF). Front. Pharmacol*.* 9, 1480 10.3389/fphar.2018.01480 30618768 PMC6304353

[B4] Chaudhuri N. Duck A. Frank R. Holme J. Leonard C. (2014). Real world experiences: pirfenidone is well tolerated in patients with idiopathic pulmonary fibrosis. Respir. Med. 108 (1), 224–226. 10.1016/j.rmed.2013.11.005 24269005

[B5] Costabel U. Bendstrup E. Cottin V. Dewint P. Egan J. J. J. Ferguson J. (2014). Pirfenidone in idiopathic pulmonary fibrosis: expert panel discussion on the management of drug-related adverse events. Adv. Ther. 31 (4), 375–391. 10.1007/s12325-014-0112-1 24639005 PMC4003341

[B6] Cottin V. Koschel D. Günther A. Albera C. Azuma A. Sköld C. M. (2018). Long-term safety of pirfenidone: results of the prospective, observational PASSPORT study. ERJ. Open. Res. 4 (4), 00084–02018. 10.1183/23120541.00084-2018 30364407 PMC6194203

[B7] Crutzen S. Schuling J. Hugtenburg J. G. Verduijn M. Teichert M. Taxis K. (2019). Development and piloting of an algorithm to select older patients for different types of medication review. Front. Pharmacol. 10, 217 10.3389/fphar.2019.00217 30941034 PMC6433968

[B8] Duck A. Pigram L. Errhalt P. Ahmed D. Chaudhuri N. (2015). IPF care: a support program for patients with idiopathic pulmonary fibrosis treated with pirfenidone in Europe. Adv. Ther. 32 (2), 87–107. 10.1007/s12325-015-0183-7 25691376 PMC4349950

[B9] Ferrara G. Luppi F. Birring S. S. Cerri S. Caminati A. Sköld M. (2018). Best supportive care for idiopathic pulmonary fibrosis: current gaps and future directions. Eur. Respir. Rev*.* 27 (147), 170076 10.1183/16000617.0076-2017 29436402 PMC9488950

[B10] Galli J. A. Pandya A. Vega-Olivo M. Dass C. Zhao H. Criner G. J. (2017). Pirfenidone and nintedanib for pulmonary fibrosis in clinical practice: tolerability and adverse drug reactions. Respirology 22 (6), 1171–1178. 10.1111/resp.13024 28317233

[B11] Homma S. Bando M. Azuma A. Sakamoto S. Sugino K. Ishii Y. (2018). Japanese guideline for the treatment of idiopathic pulmonary fibrosis. Respir. Investig. 56, 268–291. 10.1016/j.resinv.2018.03.003 29980444

[B12] Hughes G. Toellner H. Morris H. Leonard C. Chaudhuri N. (2016). Real world experiences: pirfenidone and nintedanib are effective and well tolerated treatments for idiopathic pulmonary fibrosis. J. Clin. Med. 5 (9), 78 10.3390/jcm5090078 PMC5039481 27598213

[B13] Iwata T. Yoshino I. Yoshida S. Ikeda N. Tsuboi M. Asato Y. (2016). A phase II trial evaluating the efficacy and safety of perioperative pirfenidone for prevention of acute exacerbation of idiopathic pulmonary fibrosis in lung cancer patients undergoing pulmonary resection: west Japan Oncology Group 6711 L (PEOPLE Study). Respir. Res. 17 (1), 90 10.1186/s12931-016-0398-4 27450274 PMC4957367

[B14] King T. E. Jr. Bradford W. Z. Castro-Bernardini S. Fagan E. A. Glaspole I. Glassberg M. K. (2014). A phase 3 trial of pirfenidone in patients with idiopathic pulmonary fibrosis. N. Engl. J. Med. 370, 2083–2092. 10.1056/NEJMoa1402582 24836312

[B15] Lancaster L. Albera C. Bradford W. Z. Costabel U. du Bois R. M. Fagan E. A. (2016). Safety of pirfenidone in patients with idiopathic pulmonary fibrosis: integrated analysis of cumulative data from 5 clinical trials. BMJ. Open. Respir. Res. 3 (1), e000105 10.1136/bmjresp-2015-000105 PMC4716177 26835133

[B16] Lancaster L. H. de Andrade J. A. Zibrak J. D. Padilla M. L. Albera C. Nathan S. D. (2017). Pirfenidone safety and adverse event management in idiopathic pulmonary fibrosis. Eur. Respir. Rev. 26 (146), 170057 10.1183/16000617.0057-2017 29212837 PMC9488585

[B17] Lederer D. J. Martinez F. J. (2018). Idiopathic pulmonary fibrosis. N. Engl. J. Med. 378, 1811–1823. 10.1056/NEJMra1705751 29742380

[B18] Ley B. Collard H. R. King T. E. Jr. (2011). Clinical course and prediction of survival in idiopathic pulmonary fibrosis. Am. J. Respir. Crit. Care Med. 183, 431–440. 10.1164/rccm.201006-0894CI 20935110

[B19] Martinez F. J. Flaherty K. R. (2017). Comprehensive and individualized patient care in idiopathic pulmonary fibrosis: refining approaches to diagnosis, prognosis, and treatment. Chest 151 (5), 1173–1174. 10.1016/j.chest.2017.03.017 28483105

[B20] Noble P. W. Albera C. Bradford W. Z. Costabel U. du Bois R. M. Fagan E. A. (2016). Pirfenidone for idiopathic pulmonary fibrosis: analysis of pooled data from three multinational phase 3 trials. Eur. Respir. J*.* 47 (1), 243–253. 10.1183/13993003.00026-2015 26647432 PMC4697914

[B21] Noble P. W. Albera C. Bradford W. Z. Costabel U. Glassberg M. K. Kardatzke D. (2011). Pirfenidone in patients with idiopathic pulmonary fibrosis (CAPACITY): two randomised trials. Lancet. 377 (9779), 1760–1769. 10.1016/S0140-6736(11)60405-4 21571362

[B22] Ogawa K. Miyamoto A. Hanada S. Takahashi Y. Murase K. Mochizuki S. (2018). The efficacy and safety of long-term pirfenidone therapy in patients with idiopathic pulmonary fibrosis. Intern. Med. 57 (19), 2813–2818. 10.2169/internalmedicine.0559-17 29780123 PMC6207833

[B23] Ogura T. Azuma A. Inoue Y. Taniguchi H. Chida K. Bando M. (2015). All-case post-marketing surveillance of 1371 patients treated with pirfenidone for idiopathic pulmonary fibrosis. Respir. Investig. 53 (5), 232–241. 10.1016/j.resinv.2015.06.001 26344613

[B24] Raghu G. Rochwerg B. Zhang Y. Garcia C. A. C. Azuma A. Behr J. (2015). An official ATS/ERS/JRS/ALAT clinical practice guideline: treatment of idiopathic pulmonary fibrosis. An update of the 2011 clinical practice guideline. Am. J. Respir. Crit. Care Med. 192 (2), e3-e19. 10.1164/rccm.201506-1063ST 26177183

[B25] Rajala K. Lehto J. T. Saarinen M. Sutinen E. Saarto T. Myllärniemi M. (2016). End-of-life care of patients with idiopathic pulmonary fibrosis. BMC Palliat. Care 15, 85 10.1186/s12904-016-0158-8 27729035 PMC5059981

[B26] Sköld C. M. Arnheim-Dahlström L. Bartley K. Janson C. Kirchgaessler K.-U. Levine A. (2019). Patient journey and treatment patterns in adults with IPF based on health care data in Sweden from 2001 to 2015. Respir. Med. 155, 72–78. 10.1016/j.rmed.2019.06.001 31306950

[B27] Taniguchi H. Ebina M. Kondoh Y. Ogura T. Azuma A. Suga M. (2010). Pirfenidone in patients with idiopathic pulmonary fibrosis. Eur. Respir. J. 35 (4), 821–829. 10.1183/09031936.00005209 19996196

[B28] Uehara M. Enomoto N. Oyama Y. Suzuki Y. Kono M. Furuhashi K. (2018). Body size-adjusted dose analysis of pirfenidone in patients with interstitial pneumonia. Respirology 23 (3), 318–324. 10.1111/resp.13145 28851013

[B29] Wuyts W. A. Peccatori F. A. Russell A.-M. (2014). Patient-centred management in idiopathic pulmonary fibrosis: similar themes in three communication models. Eur. Respir. Rev. 23 (132), 231–238. 10.1183/09059180.00001614 24881078 PMC9487576

[B30] Yamada K. Nabeshima T. (2015). Pharmacist-managed clinics for patient education and counseling in Japan: current status and future perspectives. J. Pharm. Health. Care. Sci. 1 (1), 2 10.1186/s40780-014-0001-4 26819713 PMC4676320

[B31] Yamamoto H. Ikesue H. Ikemura M. Miura R. Fujita K. Chung H. (2018). Evaluation of pharmaceutical intervention in direct-acting antiviral agents for hepatitis C virus infected patients in an ambulatory setting: a retrospective analysis. J. Pharm. Health. Care. Sci. 4 (1), 17 10.1186/s40780-018-0113-3 30026959 PMC6048910

[B32] Zhang J. Qian X. Zhang L. Hu L. Fan L. Wang Q. (2019). Evaluation of the effectiveness of clinical pharmacists' consultation in the treatment of infectious diseases: a single-arm, prospective cohort study. Front. Pharmacol. 10, 187 10.3389/fphar.2019.00187 30881307 PMC6405418

