# Supplementary material for: Effectiveness of Pharmacist–Physician Collaborative Management for Patients With Idiopathic Pulmonary Fibrosis Receiving Pirfenidone
Source: Front Pharmacol. 2020 Nov 26;11:529654. doi: 10.3389/fphar.2020.529654 (PMC7725709; doi:10.3389/fphar.2020.529654)
Supplement: Supplementary file 1 [file table1.docx]

**Supplementary Table S1.** Relationship between prescriptions of supportive care medications and pirfenidone discontinuation owing to adverse drug events in the conventional management group.

|  | Supportive care medication ^a^ | | *P*-value |
| --- | --- | --- | --- |
|  | Prescribed (n = 52) | Without prescription (n = 9) |  |
| Discontinuation owing to ADEs at | | | |
| 3 months | 10 (19.2%) | 3 (33.3%) | 0.386 |
| 6 months | 13 (25.0%) | 3 (33.3%) | 0.686 |
| 12 months ^b^ | 18 (35.3%) | 3 (33.3%) | 1.000 |

^a^ Supportive care medication including domperidone, metoclopramide, and mosapride.

^b^ One patient was excluded following transfer to another hospital at 11.3 months.

ADE, adverse drug reaction.
