# Supplementary figures and images for: Effectiveness of Pharmacist–Physician Collaborative Management for Patients With Idiopathic Pulmonary Fibrosis Receiving Pirfenidone
Source: Front Pharmacol. 2020 Nov 26;11:529654. doi: 10.3389/fphar.2020.529654 (PMC7725709; doi:10.3389/fphar.2020.529654)

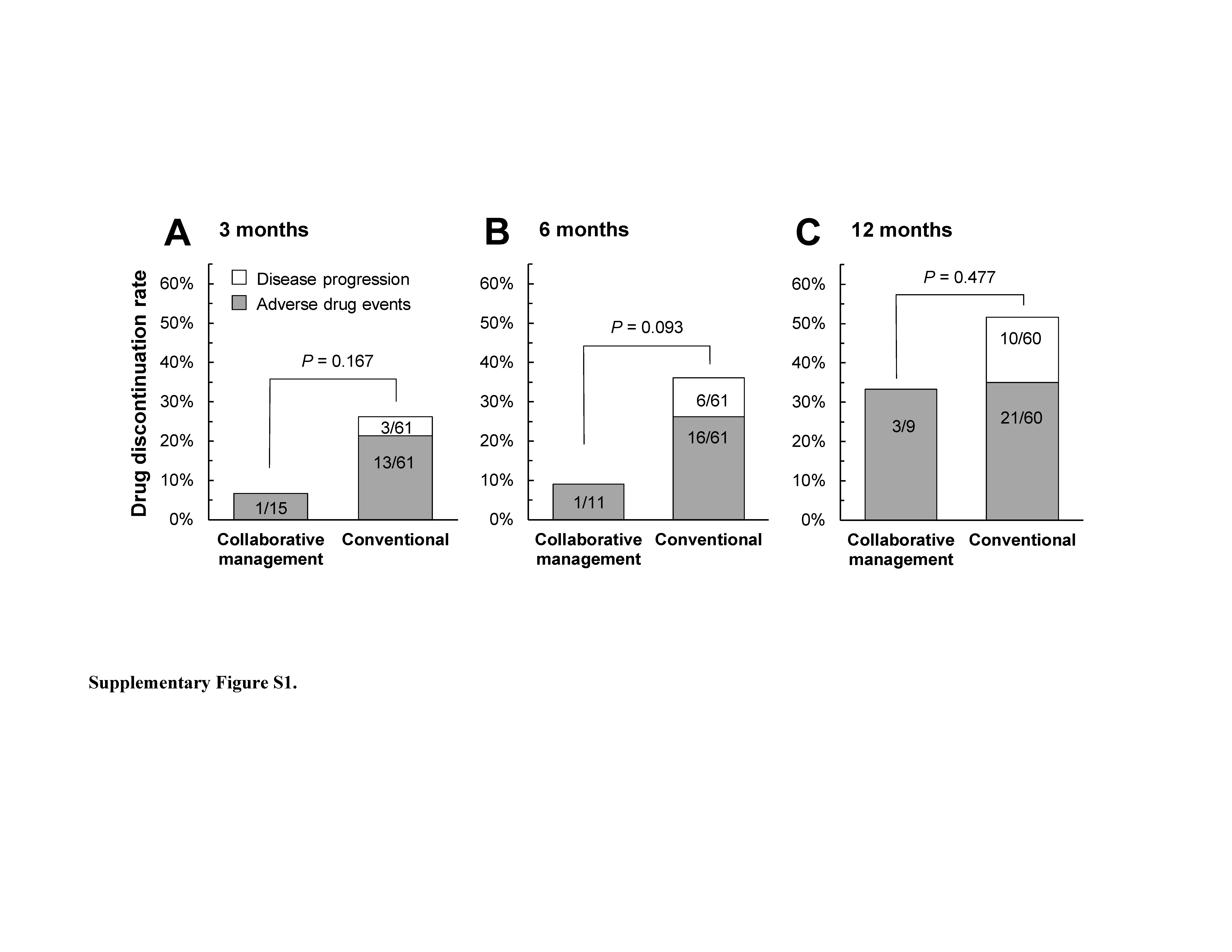

Supplement: Supplementary file 2 [file image1.tif]
